# Endometriosis burden and trends among women of childbearing age from 1990 to 2021

**DOI:** 10.3389/fendo.2025.1561673

**Published:** 2026-01-05

**Authors:** Zhongyun Tang, Chao Ma, Jin Liu, Chongdong Liu

**Affiliations:** 1 Department of Obstetrics and Gynecology, Beijing Chao-Yang Hospital, Capital Medical University, Beijing, China; 2 Department of Gynecology and Obstetrics, The Second Affiliated Hospital of Naval Medical University (Shanghai Changzheng Hospital), Shanghai, China

**Keywords:** endometriosis, GBD 2021, WCBA, disease burden, incidence, DALYs

## Abstract

**Background:**

Endometriosis is a common disease among women of childbearing age (WCBA), significantly affecting their physiological health. This study systematically analyzes the global, regional, and national burden of endometriosis in WCBA based on the Global Burden of Disease 2021 (GBD 2021) database.

**Methods:**

This study focuses specifically on the WCBA population, evaluating the burden of endometriosis in terms of incidence, prevalence, mortality, and disability-adjusted life years (DALYs).

**Results:**

Globally, the number of prevalent cases of endometriosis among WCBA increased from 19.08 million in 1990 to 21.05 million in 2021, marking a 10% rise; incident cases grew from 3.326 million to 3.439 million, an increase of 3%; DALYs rose from 1.759 million to 1.939 million, a 10% increase; and deaths increased from 21.55 to 46.72, a rise of 117%. However, the age-standardized incidence, prevalence, and DALY rates showed downward trends, with EAPCs of -0.94, -1.12, and -0.93, respectively. Regions with a middle SDI level recorded the highest numbers of prevalent cases, incident cases, DALYs, and deaths. In 2021, endometriosis exhibited marked age-related differences worldwide. Women aged 25–29 had the highest number of prevalent cases and DALYs, those aged 20–24 had the highest incidence, and those aged 45–49 had the highest number of deaths.

**Conclusion:**

The global distribution of endometriosis varies significantly across regions, highlighting the need for public health policies to be tailored to regional contexts to optimize healthcare resource allocation.

## Introduction

Endometriosis is a gynecological condition characterized by the abnormal growth of endometrial-like tissue outside the uterus, forming ectopic lesions (1–3). These lesions are responsive to hormonal fluctuations and, under the influence of the menstrual cycle, undergo proliferation and bleeding. However, due to their abnormal locations, the bleeding cannot be discharged from the body, resulting in localized inflammation, fibrosis, and tissue adhesions (4). Clinically, endometriosis primarily manifests as chronic lower abdominal pain, dysmenorrhea, irregular menstruation, and infertility (5, 6). It severely impacts patients’ quality of life and may also lead to psychological issues such as anxiety and depression (7, 8).

Endometriosis affects women of childbearing age (WCBA) across physical, psychological, and social dimensions. Pain and infertility not only compromise physical health but may also lead to emotional distress, affecting daily functioning and work productivity (5, 9). The long-term management of the disease demands significant healthcare resources and imposes economic burdens on families and society (10). In resource-limited regions, optimizing treatment strategies, improving early diagnosis rates, and enhancing patients’ quality of life remain major challenges.

This study utilizes data from the Global Burden of Disease (GBD) 2021 database to systematically review the trends and distribution of incidence, prevalence, disability-adjusted life years (DALYs), and mortality associated with endometriosis among WCBA at the global, regional, and national levels from 1990 to 2021. It also provides an in-depth analysis of disease burden across different age groups, revealing demographic and geographical heterogeneity. The aim is to provide epidemiological evidence to support public health decision-making, and to inform the optimization of prevention, diagnosis, and treatment strategies.

## Methods

### Data source and disease definition

This study is based on data from the GBD 2021 database, which provides estimated statistics for 371 diseases and injuries across 204 countries and territories from 1990 to 2021 (11–13). The Socio-Demographic Index (SDI) is a composite indicator developed by the GBD study team to measure the level of development of regions (11). Endometriosis is a chronic gynecological disorder characterized by the presence of functional endometrial tissue outside the uterine cavity and myometrium (14, 15). It is commonly found in pelvic organs and peritoneal surfaces (e.g., ovaries, uterosacral ligaments) (16, 17). Clinical manifestations include progressively worsening dysmenorrhea, abnormal menstruation, infertility, and pelvic masses. The disease is hormone-dependent and prone to recurrence. Its International Classification of Diseases (ICD) code is N80 (4, 18).

### Disability-adjusted life years and estimated annual percentage change

DALYs, a standard metric for quantifying disease burden, represent the years of healthy life lost due to illness or premature death (1). The Estimated Annual Percentage Change (EAPC) was calculated to evaluate trends in incidence, prevalence, DALYs, and mortality from 1990 to 2021, allowing assessment of epidemiological trends over fixed time intervals (19).

## Results

### Global level

**Figure 1:**
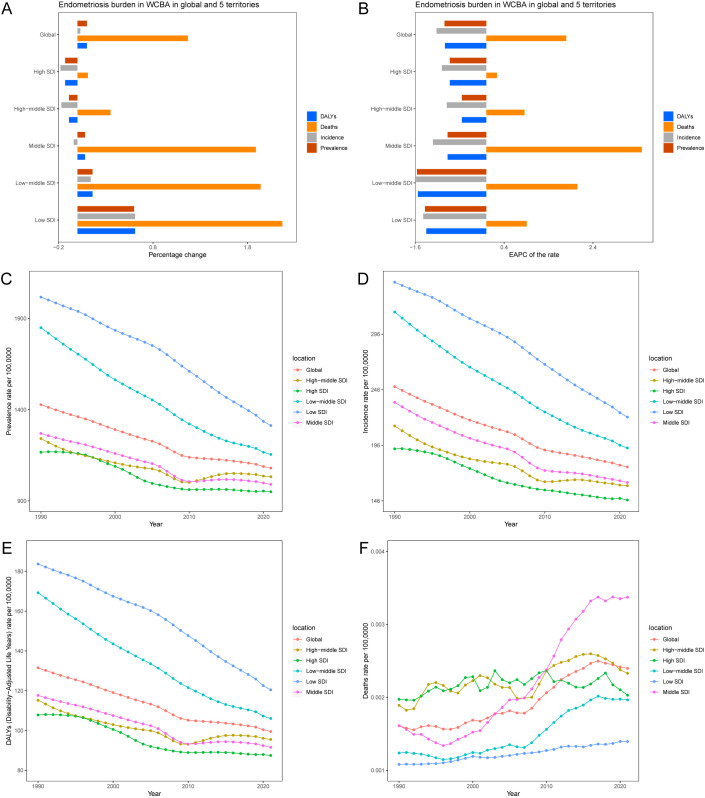
Temporal trend of endometriosis burden in WCBA in global and 5 territories. **(A)** Percentage change in cases of prevalent, incident, DALYs, and deaths in 1990 and 2021. **(B)** The EAPC of prevalence, incidence, DALY and death rates from 1990 to 2021. **(C)** The rates of prevalence from 1990 to 2021. **(D)** The rates of incidence from 1990 to 2021. **(E)** The rates of DALYs from 1990 to 2021. **(F)** The rates of death from 1990 to 2021.

Globally, the number of endometriosis prevalence cases among WCBA increased from 19.08 million in 1990 to 21.05 million in 2021, reflecting a 10% rise. In contrast, the incidence cases rose from 3,326.44 thousand in 1990 to 3,439.46 thousand in 2021, indicating a 3% increase. The DALYs cases increased from 1,758.97 thousand in 1990 to 1,939.18 thousand in 2021, a 10% rise. Mortality cases increased from 21.55 in 1990 to 46.72 in 2021, showing a 117% rise. However, from 1990 to 2021, a global downward trend was observed in the incidence, prevalence, and DALYs rates among WCBA, with EAPCs of -0.94, -1.12, and -0.93, respectively (**Figures 1A–F**, **Table 1**, **Supplementary Tables 1–3**).

**Table 1:** The prevalence of endometriosis cases and rates among WCBA in 1990 and 2021, and the trends from 1990 to 2021.

Location	Num_1990 (millions)	Num_2021 (millions)	Percentage change	Rate_1990	Rate_2021	EAPC
Andean Latin America	0.11 (0.08-0.16)	0.15 (0.1-0.21)	0.36	1173.87 (795.21-1645.35)	844.7 (575.19-1194.56)	-0.98 (-1.06–0.91)
Australasia	0.06 (0.04-0.09)	0.08 (0.05-0.11)	0.33	1209.84 (822.65-1712.09)	1057.67 (740.14-1472.86)	-0.28 (-0.37–0.19)
Caribbean	0.11 (0.07-0.15)	0.11 (0.07-0.15)	0	1134.55 (761.63-1606.34)	875.03 (590.83-1240.77)	-0.81 (-0.85–0.78)
Central Asia	0.23 (0.16-0.32)	0.27 (0.19-0.38)	0.17	1364.92 (929.59-1935.94)	1094.43 (764.72-1549.74)	-0.32 (-0.56–0.08)
Central Europe	0.31 (0.22-0.44)	0.24 (0.17-0.34)	-0.23	1021.07 (727.35-1441.28)	945.99 (664.81-1307.8)	-0.07 (-0.21-0.08)
Central Latin America	0.48 (0.32-0.69)	0.54 (0.37-0.77)	0.13	1150.09 (772.73-1635.09)	797.75 (541.02-1126.97)	-1.14 (-1.22–1.05)
Central Sub-Saharan Africa	0.24 (0.16-0.34)	0.39 (0.27-0.55)	0.63	1935.68 (1323.87-2744.29)	1204.91 (827.84-1686.88)	-1.43 (-1.56–1.3)
East Asia	3.83 (2.51-5.45)	2.78 (2-3.7)	-0.27	1147.45 (752.61-1633.98)	841.27 (602.91-1117.11)	-1.2 (-1.39–1.01)
Eastern Europe	0.97 (0.66-1.36)	0.83 (0.57-1.16)	-0.14	1754.21 (1198.88-2456.92)	1718.1 (1178.09-2402.06)	0.49 (0.25-0.74)
Eastern Sub-Saharan Africa	0.72 (0.49-1.03)	1.12 (0.75-1.59)	0.56	1664.4 (1127.28-2375.9)	1044.59 (703.38-1483.72)	-1.47 (-1.52–1.41)
Global	19.08 (13-26.68)	21.05 (14.63-29.05)	0.1	1426.95 (972.3-1995.22)	1079.91 (750.78-1490.84)	-0.94 (-0.99–0.89)
High-income Asia Pacific	0.66 (0.44-0.92)	0.49 (0.35-0.64)	-0.26	1438.59 (953.97-2008.8)	1295.42 (922.83-1689.75)	-0.48 (-0.6–0.37)
High-income North America	0.76 (0.5-1.09)	0.55 (0.41-0.72)	-0.28	1023.54 (677.3-1459.75)	656.51 (490.2-857.83)	-2.04 (-2.24–1.85)
High-middle SDI	3.45 (2.32-4.85)	3.15 (2.2-4.25)	-0.09	1241.43 (835.8-1746.32)	1032.02 (722.23-1393.02)	-0.55 (-0.66–0.45)
High SDI	2.64 (1.79-3.69)	2.31 (1.67-3.08)	-0.13	1166.62 (789.81-1629.13)	949.75 (686.18-1264.88)	-0.82 (-0.92–0.72)
Low-middle SDI	5.05 (3.44-7.09)	5.84 (4.03-8.18)	0.16	1849.17 (1260.79-2598.13)	1154.39 (795.29-1615.93)	-1.56 (-1.59–1.52)
Low SDI	2.25 (1.53-3.16)	3.6 (2.43-5.07)	0.6	2017.03 (1369.61-2827.66)	1312.83 (886.48-1847.05)	-1.38 (-1.45–1.3)
Middle SDI	5.68 (3.79-7.96)	6.12 (4.27-8.49)	0.08	1269.52 (847.75-1781.52)	990.35 (690.89-1372.31)	-0.87 (-0.95–0.79)
North Africa and Middle East	1.46 (0.99-2.05)	2.11 (1.46-2.97)	0.45	1867.26 (1262.32-2628.69)	1324.57 (915.92-1865.46)	-1.11 (-1.18–1.05)
Oceania	0.03 (0.02-0.05)	0.07 (0.04-0.09)	1.33	2181.61 (1468.82-3096.1)	1873.14 (1255.06-2630.71)	-0.5 (-0.51–0.48)
South Asia	4.77 (3.23-6.68)	5.64 (3.89-7.9)	0.18	1870.61 (1265.37-2620.5)	1140.97 (786.29-1598.03)	-1.65 (-1.69–1.62)
Southeast Asia	1.86 (1.29-2.59)	2.28 (1.62-3.14)	0.23	1549.45 (1071.2-2159.08)	1246.1 (882.42-1716.43)	-0.64 (-0.68–0.61)
Southern Latin America	0.12 (0.08-0.17)	0.15 (0.11-0.19)	0.25	975.42 (653.87-1353.62)	851.43 (622.81-1112.37)	-0.3 (-0.43–0.17)
Southern Sub-Saharan Africa	0.19 (0.13-0.26)	0.25 (0.17-0.35)	0.32	1408.73 (960.96-1976.34)	1149.86 (780.3-1613.34)	-0.57 (-0.63–0.51)
Tropical Latin America	0.45 (0.29-0.67)	0.59 (0.4-0.82)	0.31	1139.61 (734.02-1674.2)	967.23 (664.88-1357.6)	-0.98 (-1.23–0.73)
Western Europe	0.91 (0.62-1.28)	0.84 (0.6-1.12)	-0.08	954.95 (648.13-1344.42)	899.84 (648.95-1206.69)	-0.13 (-0.15–0.11)
Western Sub-Saharan Africa	0.81 (0.55-1.14)	1.58 (1.06-2.2)	0.95	1849.74 (1258.27-2603.44)	1313.79 (886.76-1834.28)	-1.04 (-1.11–0.97)

### SDI regional level

**Figure 2:**
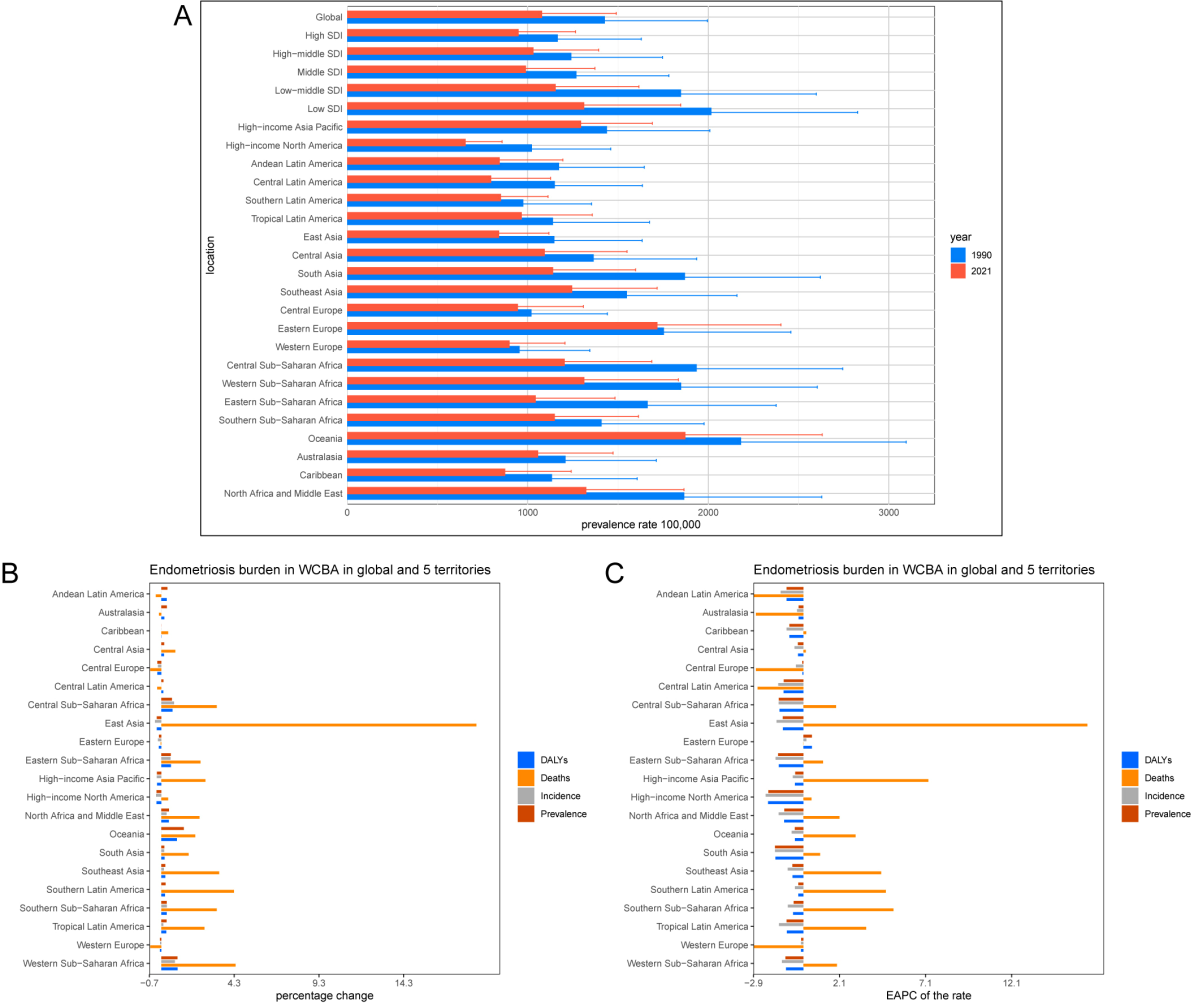
Temporal trend of endometriosis burden in WCBA in regions. **(A)** Prevalence rate per 100,000 population in 1990 and 2021. **(B)** Percentage change in cases of prevalent, incident, DALYs, and death in 1990 and 2021. **(C)** EAPC of rates of prevalent, incident, DALYs and death from 1990 to 2021.

In 2021, the middle SDI regions had the highest numbers of prevalence, incidence, DALYs, and death cases, with 6.12 million, 1,005.31 thousand, 566.31 thousand, and 20.86 cases, respectively, representing percentage changes of 8%, -4%, 8%, and 189%. The highest prevalence, incidence, and DALYs rates were observed in the low SDI regions, with 1,312.83, 221.39, and 120.44 per 100,000 population, and corresponding EAPCs of -1.38, -1.42, and -1.35 (**Figure 2A**, **Supplementary Figures 1, 2**). However, the highest death rate was found in the middle SDI regions, at 0.0034 per 100,000, with an EAPC of 3.5 (**Supplementary Figure 3**).

### GBD regional level

Over the past 32 years, the fastest increase in prevalence cases occurred in Oceania, with a percentage change of 133%. The fastest increases in incidence and DALYs cases were observed in Western Sub-Saharan Africa, with percentage changes of 86% and 96%, respectively. The highest increase in death cases occurred in East Asia, with a rise of 1859%. The highest prevalence, incidence, and DALYs rates were also recorded in Oceania, at 1,873.14, 302.82, and 172.76 per 100,000 population, with EAPCs of -0.5, -0.68, and -0.49, respectively. The highest death rate was found in Tropical Latin America at 0.015 per 100,000, with an EAPC of 3.65. The fastest-growing prevalence, incidence, and DALYs rates were observed in Eastern Europe, with EAPCs of 0.49, 0.17, and 0.49, respectively. The highest growth rate in death rate was recorded in the High-income Asia Pacific region, with an EAPC of 7.26 (**Figures 2B, C**).

### Country level

**Figure 3:**
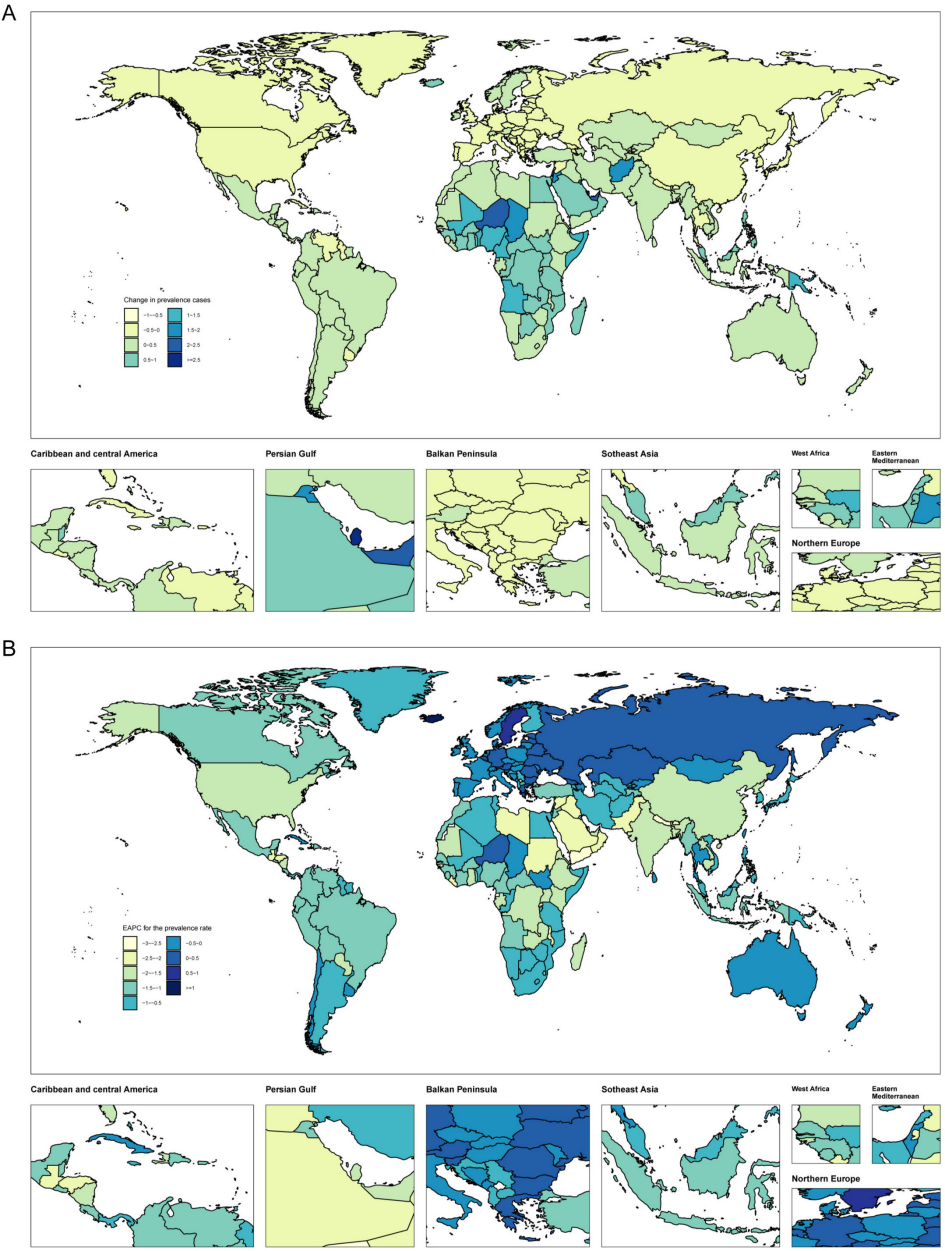
Temporal trend of endometriosis burden in WCBA globally. **(A)** Percentage change in prevalent cases across 204 countries in 1990 and 2021. **(B)** EAPC in prevalent rates across 204 countries from 1990 to 2021.

From 1990 to 2021, about 64% of countries experienced increasing trends in prevalence and DALYs cases. The countries with the fastest-growing prevalence cases included Qatar, Niger, and the United Arab Emirates, with percentage changes of 364%, 224%, and 203%, respectively. The fastest-growing incidence cases were seen in Qatar (319%), Niger (186%), and the United Arab Emirates (186%). DALYs cases increased most rapidly in Qatar (363%), Niger (227%), and the United Arab Emirates (200%). The fastest rise in death cases occurred in China, Malaysia, and Egypt, with percentage increases of 2013%, 1150%, and 800%, respectively. The countries with the fastest-growing prevalence, incidence, and DALYs rates were Iceland, with EAPCs of 1.2, 2.23, and 1.2, respectively (**Figures 3A, B**, **Supplementary Figures 4–9**, **Supplementary Table 4–7**).

### Age patterns

**Figure 4:**
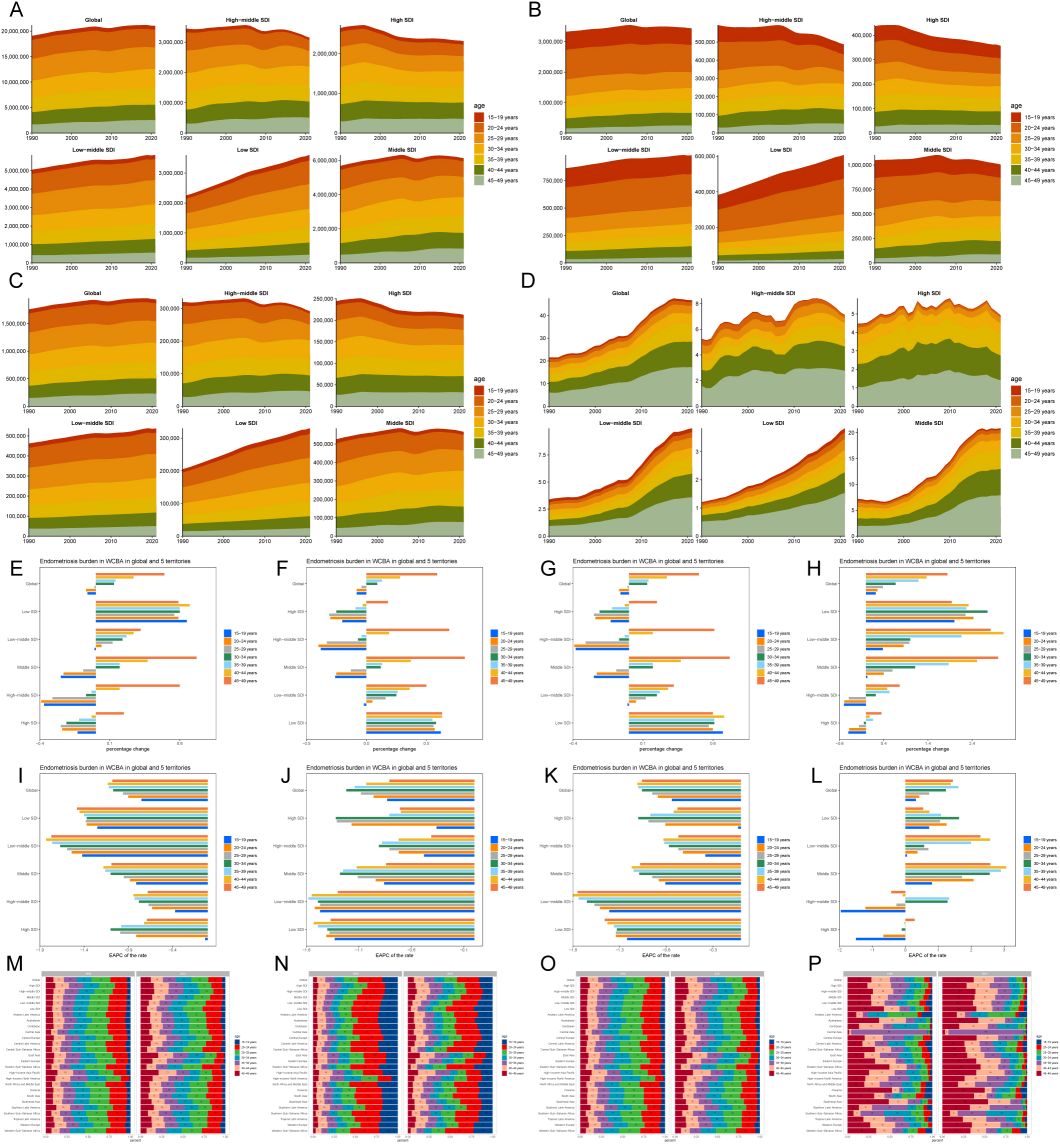
Temporal trend of endometriosis burden in WCBA by age pattern in different regions. Prevalent **(A)**, incident **(B)**, DALYs **(C)**, and death **(D)** cases of 7 age groups (15–49 years, 5-year intervals) from 1990 to 2021 globally and in 5 territories (low to high SDI). Percentage change in prevalent **(E)**, incident **(F)**, DALYs **(G)**, and death **(H)** cases of 7 age groups globally and in 5 territories in 1990 and 2021. EAPC of prevalent **(I)**, incident **(J)**, DALYs **(K)**, and death **(L)** rates of 7 age groups globally and in 5 territories from 1990 to 2021. The distribution of prevalent **(M)**, incident **(N)**, DALYs **(O)**, and death **(P)** cases across 7 age groups as percentages globally, in 5 territories, and 21 GBD regions in 1990 and 2021.

In 2021, the age group with the highest prevalence and DALYs cases of endometriosis globally was 25–29 years, with 4,229.82 and 391.77 cases, respectively. The highest incidence cases were observed in the 20–24 age group (893.93), while the highest number of death cases was seen in the 45–49 age group (17.22). The 45–49 age group also showed the most rapid increase in prevalence, incidence, DALYs, and death cases, with percentage changes of 49%, 59%, 50%, and 184%, respectively. The highest prevalence and DALYs rates were in the 25–29 age group, with EAPCs of -0.96 and -0.95, respectively. The incidence rate was highest in the 20–24 age group, with an EAPC of -0.96. Additionally, in the middle SDI region, the 45–49 age group experienced the fastest increases in prevalence, incidence, and DALYs cases, with percentage changes of 72%, 82%, and 72%, respectively. The 40–44 age group in low-middle SDI regions showed the most rapid increase in death cases, with a percentage change of 311% (**Figures 4A–L**, **Table 2**, **Supplementary Table 8–10**).

**Table 2:** The prevalence of endometriosis cases and rates among WCBA in 1990 and 2021, and the trends in age patterns from 1990 to 2021.

Location	Age	Num_1990 (thousands)	Num_2021 (thousands)	Percentage change	Rate_1990	Rate_2021	EAPC
Global	15–19 years	808.46 (497.19-1298.94)	758.18 (472.05-1214.67)	-0.06 (-0.05–0.06)	316.37 (194.57-508.31)	249.69 (155.46-400.02)	-0.75 (-0.8–0.7)
Global	15–49 years	19083.47 (13003.2-26683.3)	21045.89 (14631.68-29054.37)	0.1 (0.13-0.09)	1426.95 (972.3-1995.22)	1079.91 (750.78-1490.84)	-0.94 (-0.99–0.89)
Global	20–24 years	3734.1 (2115.48-5834.9)	3477.85 (1989.29-5397.95)	-0.07 (-0.06–0.07)	1529.52 (866.52-2390.03)	1183.94 (677.2-1837.59)	-0.9 (-0.97–0.83)
Global	25–29 years	4260 (2646.56-6584.28)	4229.82 (2666.96-6401.55)	-0.01 (0.01–0.03)	1935.49 (1202.44-2991.5)	1453.61 (916.52-2199.94)	-0.96 (-1.01–0.91)
Global	30–34 years	3325.35 (1993.03-5184.03)	3743.67 (2298.4-5797.5)	0.13 (0.15-0.12)	1749.18 (1048.36-2726.87)	1252.35 (768.87-1939.41)	-1.07 (-1.11–1.03)
Global	35–39 years	2886.03 (1740.31-4385.56)	3297.24 (2047.32-4939.55)	0.14 (0.18-0.13)	1663.87 (1003.33-2528.38)	1186.9 (736.97-1778.08)	-1.12 (-1.18–1.05)
Global	40–44 years	2382.13 (1381.39-3615.34)	3019.27 (1756.56-4519.72)	0.27 (0.27-0.25)	1698.79 (985.12-2578.23)	1217.01 (708.03-1821.81)	-1.13 (-1.2–1.06)
Global	45–49 years	1687.4 (1105.54-2437.28)	2519.85 (1643.75-3621.3)	0.49 (0.49-0.49)	1482.77 (971.47-2141.71)	1069.35 (697.56-1536.77)	-1.08 (-1.14–1.01)
Low SDI	15–19 years	109.16 (67.89-169.16)	180.38 (111.62-281.26)	0.65 (0.64-0.66)	434.39 (270.18-673.18)	292.55 (181.04-456.16)	-1.25 (-1.31–1.2)
Low SDI	15–49 years	2252.73 (1529.65-3158.08)	3601.25 (2431.71-5066.66)	0.6 (0.59-0.6)	2017.03 (1369.61-2827.66)	1312.83 (886.48-1847.05)	-1.38 (-1.45–1.3)
Low SDI	20–24 years	493.61 (286.16-769.67)	784.7 (440.5-1233.21)	0.59 (0.54-0.6)	2272.54 (1317.44-3543.48)	1488.07 (835.36-2338.63)	-1.37 (-1.45–1.3)
Low SDI	25–29 years	534.88 (338.08-814.64)	836.19 (513.55-1275.12)	0.56 (0.52-0.57)	2887.57 (1825.16-4397.92)	1898.32 (1165.85-2894.77)	-1.38 (-1.46–1.29)
Low SDI	30–34 years	388.76 (226.92-614.75)	622.89 (362.16-974.01)	0.6 (0.6-0.58)	2555.81 (1491.8-4041.5)	1678.11 (975.68-2624.06)	-1.37 (-1.46–1.28)
Low SDI	35–39 years	311.39 (186.93-476.58)	497.69 (299.61-757.85)	0.6 (0.6-0.59)	2416.77 (1450.79-3698.79)	1561.84 (940.23-2378.27)	-1.4 (-1.48–1.32)
Low SDI	40–44 years	243.96 (138.3-366.7)	407.72 (233.83-613.51)	0.67 (0.69-0.67)	2460.44 (1394.78-3698.4)	1560.96 (895.2-2348.82)	-1.45 (-1.52–1.39)
Low SDI	45–49 years	170.97 (113.57-241.72)	271.68 (180.15-390.55)	0.59 (0.59-0.62)	2059.46 (1368.08-2911.69)	1308.21 (867.48-1880.59)	-1.48 (-1.55–1.42)
Low-middle SDI	15–19 years	229.87 (143.52-358.54)	228.31 (140.84-355.87)	-0.01 (-0.02–0.01)	391.25 (244.28-610.27)	252.55 (155.79-393.65)	-1.42 (-1.45–1.4)
Low-middle SDI	15–49 years	5046.68 (3440.89-7090.71)	5844.34 (4026.3-8180.96)	0.16 (0.17-0.15)	1849.17 (1260.79-2598.13)	1154.39 (795.29-1615.93)	-1.56 (-1.59–1.52)
Low-middle SDI	20–24 years	1072.12 (613.22-1690.4)	1119.64 (633.45-1760.87)	0.04 (0.03-0.04)	2055.66 (1175.77-3241.14)	1286.81 (728.03-2023.78)	-1.54 (-1.57–1.51)
Low-middle SDI	25–29 years	1168.04 (735.36-1792.32)	1307.88 (833.01-1986.53)	0.12 (0.13-0.11)	2602.43 (1638.41-3993.34)	1609.76 (1025.28-2445.06)	-1.59 (-1.63–1.56)
Low-middle SDI	30–34 years	862.87 (507.56-1360.58)	1028.3 (624.28-1622.94)	0.19 (0.23-0.19)	2304.45 (1355.52-3633.65)	1391.33 (844.67-2195.89)	-1.67 (-1.71–1.63)
Low-middle SDI	35–39 years	703.92 (422.61-1069.96)	856.01 (520.04-1287.72)	0.22 (0.23-0.2)	2197.41 (1319.24-3340.06)	1285.56 (781-1933.9)	-1.76 (-1.8–1.72)
Low-middle SDI	40–44 years	587.71 (336.81-875.75)	747.2 (431.83-1137.48)	0.27 (0.28-0.3)	2265.24 (1298.21-3375.48)	1295.38 (748.64-1972)	-1.83 (-1.86–1.79)
Low-middle SDI	45–49 years	422.16 (281.3-595.68)	557 (364.84-801.81)	0.32 (0.3-0.35)	1944.97 (1296-2744.42)	1126.7 (737.98-1621.88)	-1.77 (-1.8–1.74)
Middle SDI	15–19 years	240.56 (142.82-384.76)	181.02 (111.64-286.13)	-0.25 (-0.22–0.26)	260.9 (154.9-417.29)	206.09 (127.1-325.74)	-0.81 (-0.89–0.74)
Middle SDI	15–49 years	5675.58 (3790-7964.51)	6125 (4272.93-8487.31)	0.08 (0.13-0.07)	1269.52 (847.75-1781.52)	990.35 (690.89-1372.31)	-0.87 (-0.95–0.79)
Middle SDI	20–24 years	1188.06 (663.34-1870.51)	913.95 (518.32-1431.95)	-0.23 (-0.22–0.23)	1344.99 (750.96-2117.59)	1054.55 (598.05-1652.24)	-0.88 (-0.98–0.78)
Middle SDI	25–29 years	1303.18 (812.17-2013.01)	1186.84 (749.57-1803.32)	-0.09 (-0.08–0.1)	1739.71 (1084.22-2687.31)	1310 (827.35-1990.45)	-0.95 (-1.03–0.88)
Middle SDI	30–34 years	948.74 (565.37-1486.44)	1109.73 (676.77-1727.53)	0.17 (0.2-0.16)	1580.2 (941.67-2475.79)	1123.74 (685.32-1749.34)	-1.1 (-1.17–1.03)
Middle SDI	35–39 years	839.66 (493.91-1292.55)	982.51 (604.28-1479.65)	0.17 (0.22-0.14)	1514.78 (891.03-2331.8)	1072.05 (659.35-1614.5)	-1.16 (-1.25–1.06)
Middle SDI	40–44 years	671.1 (392.04-1033.62)	918.26 (541.48-1388.49)	0.37 (0.38-0.34)	1588.17 (927.76-2446.09)	1121.87 (661.55-1696.37)	-1.18 (-1.28–1.08)
Middle SDI	45–49 years	484.27 (311.04-700.35)	832.69 (530.42-1207.05)	0.72 (0.71-0.72)	1428.82 (917.7-2066.36)	1026.6 (653.95-1488.14)	-1.09 (-1.17–1.02)
High-middle SDI	15–19 years	125.29 (76.45-204.75)	78.53 (48.71-126.71)	-0.37 (-0.36–0.38)	264.54 (161.43-432.32)	228.07 (141.44-367.98)	-0.37 (-0.53–0.22)
High-middle SDI	15–49 years	3448.11 (2321.48-4850.47)	3148.99 (2203.72-4250.5)	-0.09 (-0.05–0.12)	1241.43 (835.8-1746.32)	1032.02 (722.23-1393.02)	-0.55 (-0.66–0.45)
High-middle SDI	20–24 years	597.09 (326.55-944.59)	366.04 (215.78-556.94)	-0.39 (-0.34–0.41)	1240.29 (678.31-1962.13)	1028.33 (606.2-1564.62)	-0.63 (-0.73–0.52)
High-middle SDI	25–29 years	747.64 (466.18-1156.67)	516.78 (332.65-767.97)	-0.31 (-0.29–0.34)	1634.33 (1019.06-2528.48)	1282.04 (825.26-1905.2)	-0.67 (-0.75–0.59)
High-middle SDI	30–34 years	636.48 (376.62-998.02)	594.61 (372.33-915.91)	-0.07 (-0.01–0.08)	1524.56 (902.11-2390.55)	1154.78 (723.11-1778.79)	-0.78 (-0.88–0.67)
High-middle SDI	35–39 years	575.59 (344.73-883.47)	560.13 (349.3-830.66)	-0.03 (0.01–0.06)	1457.31 (872.8-2236.82)	1130.41 (704.93-1676.37)	-0.83 (-0.96–0.7)
High-middle SDI	40–44 years	451.81 (264.1-690.88)	529.21 (314.06-788.34)	0.17 (0.19-0.14)	1469.56 (859.04-2247.2)	1162.61 (689.95-1731.87)	-0.84 (-0.97–0.71)
High-middle SDI	45–49 years	314.22 (202.74-462.54)	503.69 (325.94-724.65)	0.6 (0.61-0.57)	1281.61 (826.92-1886.6)	1044.39 (675.83-1502.55)	-0.68 (-0.75–0.6)
High SDI	15–19 years	102.89 (58.61-184.5)	89.34 (48.51-164.33)	-0.13 (-0.17–0.11)	322.85 (183.91-578.92)	307.09 (166.76-564.86)	-0.03 (-0.13-0.07)
High SDI	15–49 years	2644.81 (1790.55-3693.36)	2309.39 (1668.5-3075.66)	-0.13 (-0.07–0.17)	1166.62 (789.81-1629.13)	949.75 (686.18-1264.88)	-0.82 (-0.92–0.72)
High SDI	20–24 years	380.12 (215.7-603.47)	290.74 (167.89-441.08)	-0.24 (-0.22–0.27)	1132.04 (642.37-1797.2)	922.31 (532.58-1399.21)	-0.81 (-0.9–0.72)
High SDI	25–29 years	502.76 (313.59-800.79)	378.71 (244.54-573.4)	-0.25 (-0.22–0.28)	1402.56 (874.83-2233.98)	1095.82 (707.6-1659.18)	-0.99 (-1.08–0.9)
High SDI	30–34 years	485.74 (290.58-746.3)	385.19 (245.21-575.9)	-0.21 (-0.16–0.23)	1368.86 (818.9-2103.16)	1028.99 (655.05-1538.46)	-1.1 (-1.2–1)
High SDI	35–39 years	453.22 (271.19-686.37)	398.27 (254.03-580.21)	-0.12 (-0.06–0.15)	1355.35 (811-2052.58)	1049.94 (669.69-1529.59)	-0.98 (-1.07–0.88)
High SDI	40–44 years	425.68 (248.79-656.62)	414.4 (246.64-597.47)	-0.03 (-0.01–0.09)	1363.36 (796.82-2103)	1128.79 (671.84-1627.48)	-0.72 (-0.81–0.63)
High SDI	45–49 years	294.4 (190.65-435.57)	352.75 (237.43-497.19)	0.2 (0.25-0.14)	1165.18 (754.55-1723.89)	982.44 (661.27-1384.71)	-0.69 (-0.8–0.57)

In 2021, the highest proportions of global prevalence, incidence, and DALYs cases were in the 25–29, 20–24, and 25–29 age groups, respectively—each showing a decrease of 2%, 3%, and 2% compared to 1990. The death cases were most concentrated in the 45–49 age group, increasing by 9% compared to 1990 (**Figures 4M–P**).

### The association between endometriosis burden and SDI

**Figure 5:**
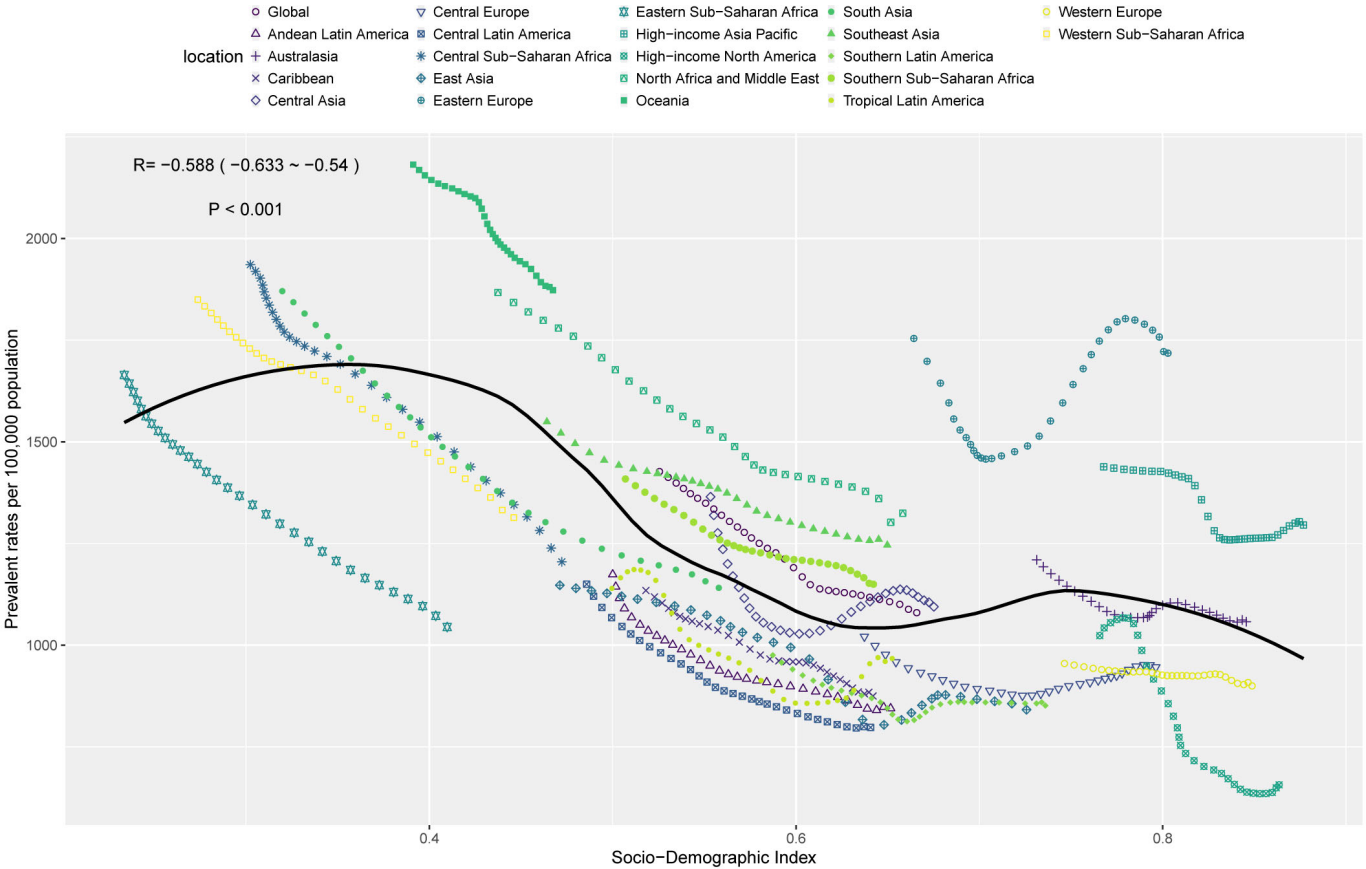
The associations between the SDI and prevalent rates per 100,000 population of endometriosis in WCBA across 21 GBD regions.

In 2021, prevalence, incidence, and DALYs rates of endometriosis were significantly negatively correlated with the SDI, indicating that as the SDI level increased, the disease burden decreased significantly (**Figure 5**, **Supplementary Figures 10, 11**). However, the death rate showed a significant positive correlation with SDI. Specifically, when the SDI ranged between 0.4 and 0.7, death rates increased with rising SDI levels (**Supplementary Figure 12**). Notably, some regions such as Oceania, Eastern Europe, and High-income Asia Pacific exhibited a disease burden significantly higher than expected, while regions including East Asia, Western Europe, and Central Latin America showed a significantly lower-than-expected disease burden, warranting further investigation.

## Discussion

Globally, while the number of endometriosis prevalence cases among WCBA increased from 1990 to 2021, the prevalence, incidence, and DALY rates showed declining trends. These changes may be associated with changes in lifestyle, environmental factors, advances in diagnostic technologies, and enhanced preventive measures (20). However, during the same period, the number of deaths increased significantly, which may be attributed to delayed diagnosis and treatment, as well as population aging.

At the SDI regional level, in 2021, regions with a middle SDI had the highest numbers of prevalence, incidence, DALYs, and deaths due to endometriosis. In contrast, regions with a low SDI had the highest prevalence, incidence, and DALY rates. At the GBD regional level, the most rapid increase in the number of prevalent cases over the past 32 years occurred in Oceania, while the fastest increases in incidence and DALYs were observed in sub-Saharan Africa, and the most significant rise in deaths occurred in East Asia. These findings highlight the marked geographic heterogeneity in the burden of endometriosis, suggesting that public health policies should be tailored to regional contexts in order to optimize healthcare resource allocation (21).

From 1990 to 2021, approximately 64% of countries experienced an increasing trend in prevalence and DALY cases. Qatar, Niger, and the United Arab Emirates showed the fastest increases in prevalence, incidence, and DALY cases. Iceland recorded the fastest growth in prevalence, incidence, and DALY rates. This upward trend may be driven by multiple factors. Economic development and lifestyle changes are among the key contributors. For instance, as some countries undergo rapid economic growth, lifestyles have become increasingly Westernized, with higher-calorie diets and decreased physical activity leading to increased obesity rates and a rise in chronic disease prevalence. Demographic changes, including aging populations and shifts in the proportion of younger age groups, may also contribute to rising disease incidence and mortality (22).

In 2021, the burden of endometriosis demonstrated clear age-related differences. Women aged 20–24 had the highest number of prevalent cases, which may be related to physiological characteristics such as regular menstruation and robust ovarian function. Women aged 25–29 had the highest number of incident cases and DALYs, likely because the disease tends to worsen with age, and this group represents the peak reproductive period when the impact of the disease is more pronounced. Middle-aged women (45–49 years) had the highest number of deaths and experienced a rapid increase in mortality. This may be related to declining physiological function, weakened immune response, and the potential co-existence of other chronic conditions. Therefore, there is a need to strengthen health education and screening programs for middle-aged and older women, and to implement relevant public health policies to enable timely detection and effective treatment.

This study, while leveraging a comprehensive dataset from the GBD 2021, is subject to several important limitations that warrant consideration. First, the reliability of the findings depends on the quality and consistency of reporting across different countries, as variations in healthcare infrastructure, diagnostic capabilities, and data collection methodologies may introduce bias and affect the accuracy of estimates. Second, the GBD definition of endometriosis may not fully capture undiagnosed or subclinical cases, potentially limiting a comprehensive understanding of the disease burden.

## Conclusion

Using data from the GBD 2021 database, this study systematically analyzed the burden of endometriosis among WCBA at global, regional, and national levels from 1990 to 2021. The findings reveal that although the absolute number of prevalent cases, incident cases, DALYs, and deaths due to endometriosis in WCBA has increased, the prevalence, incidence, and DALY rates have declined. These trends may be linked to lifestyle changes, environmental factors, improvements in diagnostic technologies, and strengthened preventive efforts. However, the significant rise in mortality may be associated with delayed diagnosis and treatment, as well as population aging. The burden of endometriosis varies markedly across SDI regions and age groups, underscoring the need for public health strategies tailored to specific regional circumstances. Optimizing healthcare resource allocation and improving early diagnosis rates are essential to enhance the quality of life for affected individuals.

## Data Availability

The original contributions presented in the study are included in the article/**Supplementary Material**. Further inquiries can be directed to the corresponding author.
