# Correction: Endometriosis burden and trends among women of childbearing age from 1990 to 2021

**DOI:** 10.3389/fendo.2026.1784098

**Published:** 2026-01-20

**Authors:** Zhongyun Tang, Chao Ma, Jin Liu, Chongdong Liu

**Affiliations:** 1 Department of Obstetrics and Gynecology, Beijing Chao-Yang Hospital, Capital Medical University, Beijing, China; 2 Department of Gynecology and Obstetrics, The Second Affiliated Hospital of Naval Medical University (Shanghai Changzheng Hospital), Shanghai, China

**Keywords:** endometriosis, GBD 2021, WCBA, disease burden, incidence, DALYs

Affiliation “Department of Obstetrics and Gynecology, Beijing Chao-Yang Hospital, Capital Medical University, Beijing, China.” was erroneously given as “Department of Obstetrics and Gynecology, Jiading District Central Hospital Affiliated Shanghai University of Medicine and Health Sciences, Shanghai, China”.

The original version of this article has been updated.

Author “Chao Ma” was erroneously assigned as corresponding author. The correct corresponding author is “Chongdong Liu”.

The original version of this article has been updated.

In the published article, there was a mistake in the **Funding** statement. The original **Funding** statement read: “The author(s) declared that financial support was received for this work and/or its publication. This work was supported by the Cervical Disease Specialist Department, Key Project of the Clinical Specialty Competence Enhancement Program (JZXLCZK-2024-01).”

The correct **Funding** statement reads:

“The author(s) declared that financial support was not received for this work and/or its publication.”

The original version of this article has been updated.

