# Supplementary material for: Endometriosis burden and trends among women of childbearing age from 1990 to 2021
Source: Front Endocrinol (Lausanne). 2026 Jan 5;16:1561673. doi: 10.3389/fendo.2025.1561673 (PMC12813029; doi:10.3389/fendo.2025.1561673)
Supplement: Supplementary file 1 [file DataSheet1.docx]

Supplementary Figure


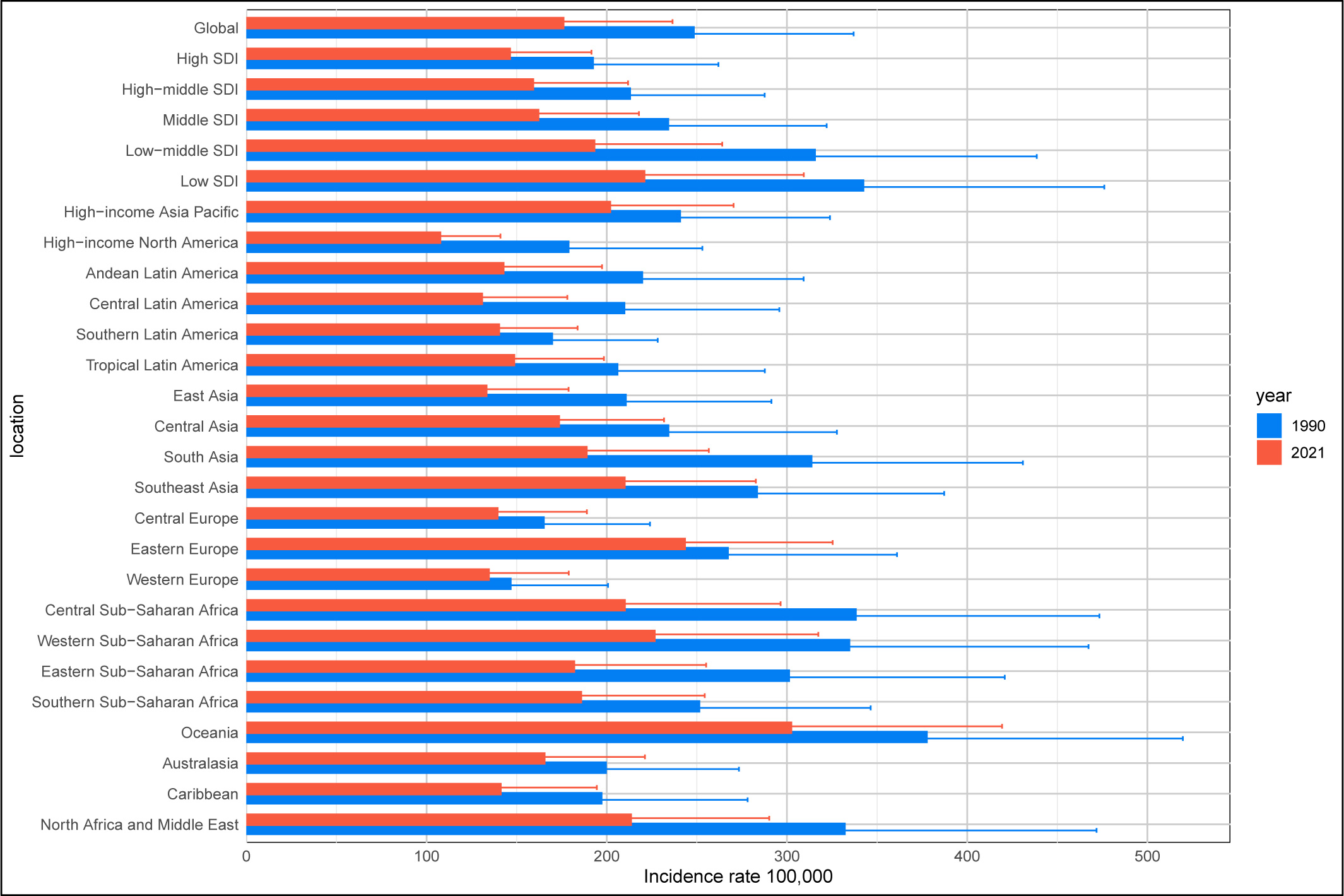


Supplementary Figure 1. Incidence rate per 100,000 population in 1990 and 2021.


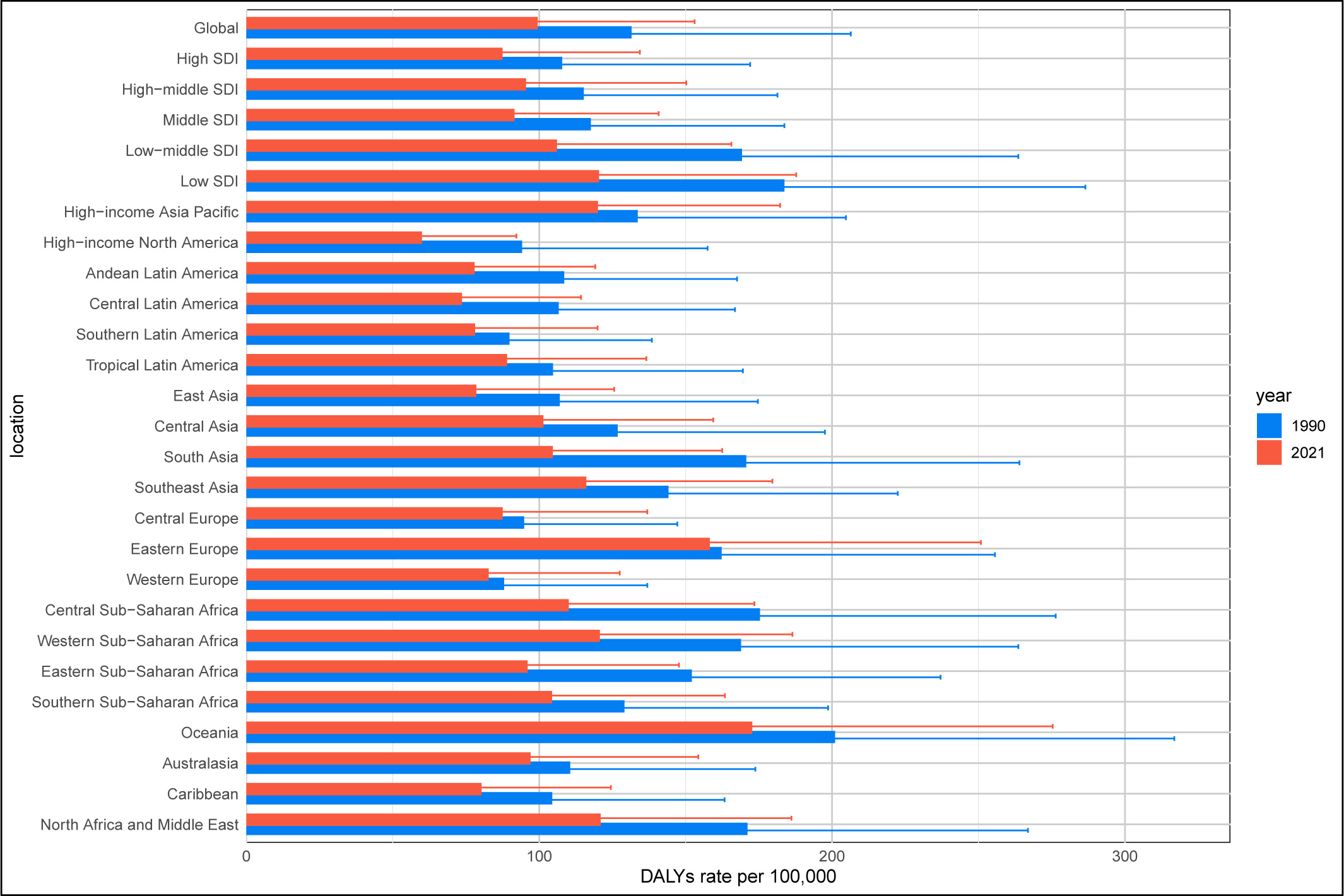


Supplementary Figure 2. DALYs rate per 100,000 population in 1990 and 2021.


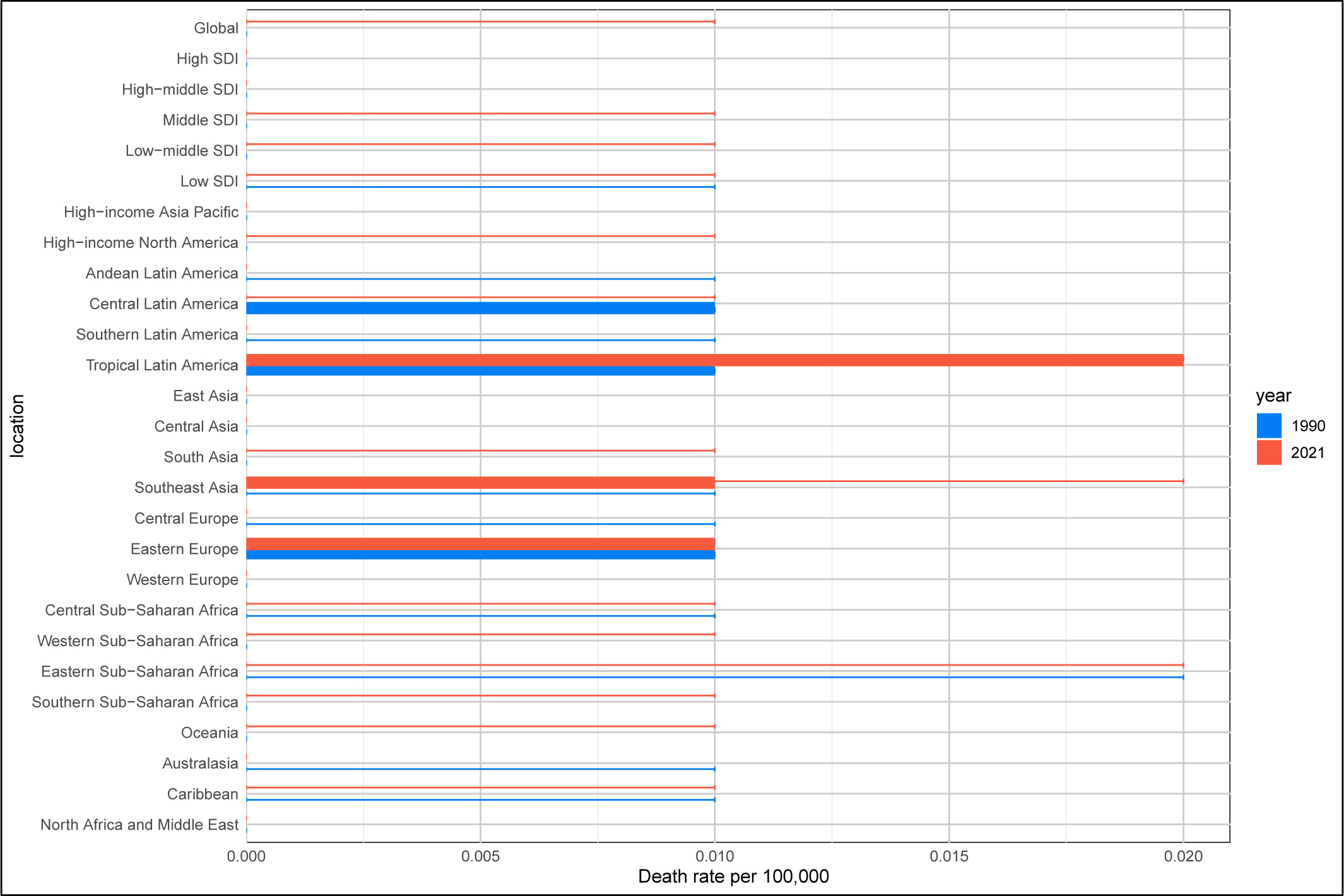


Supplementary Figure 3. Death rate per 100,000 population in 1990 and 2021.


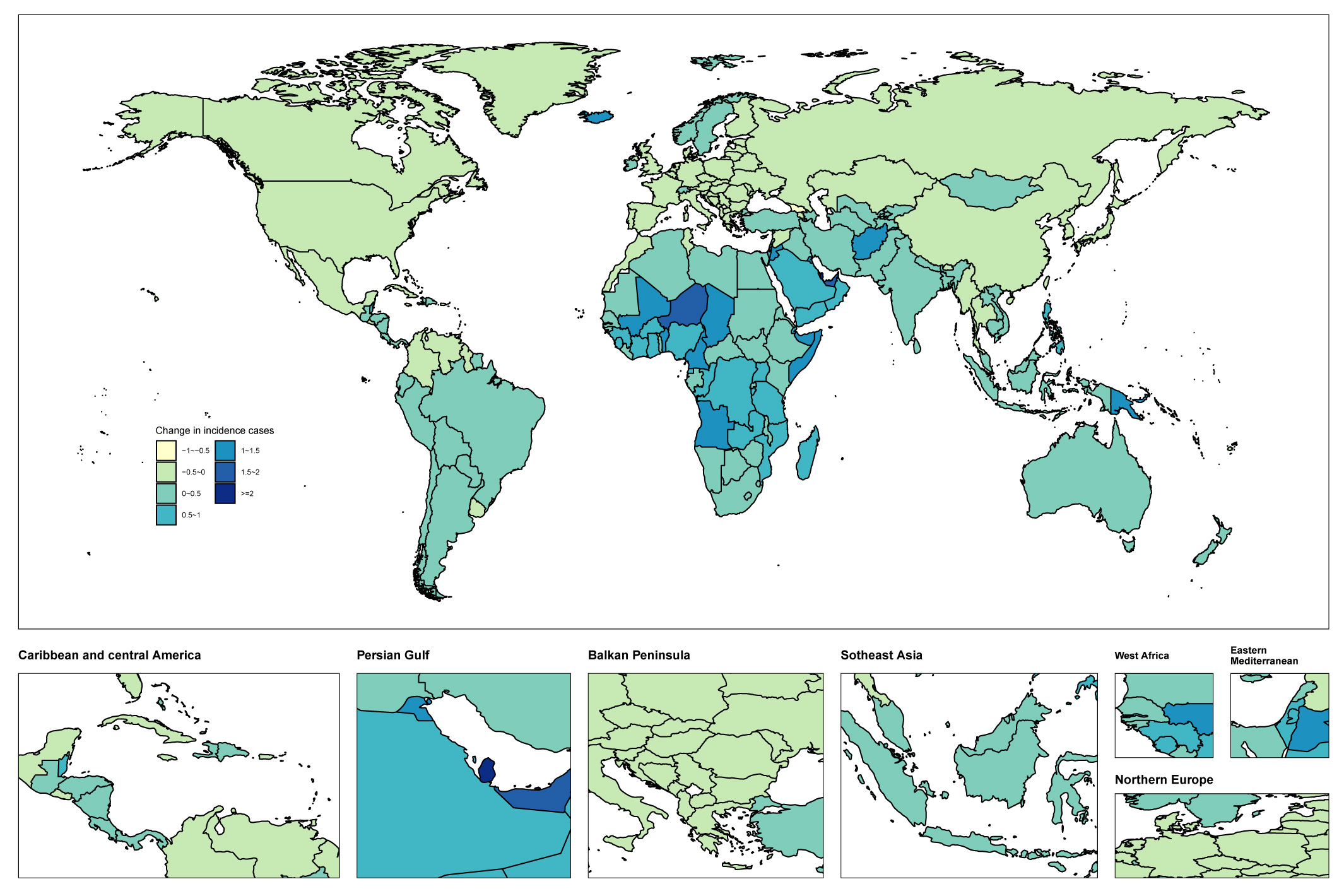


Supplementary Figure 4. Percentage change in incidence cases across 204 countries in 1990 and 2021.


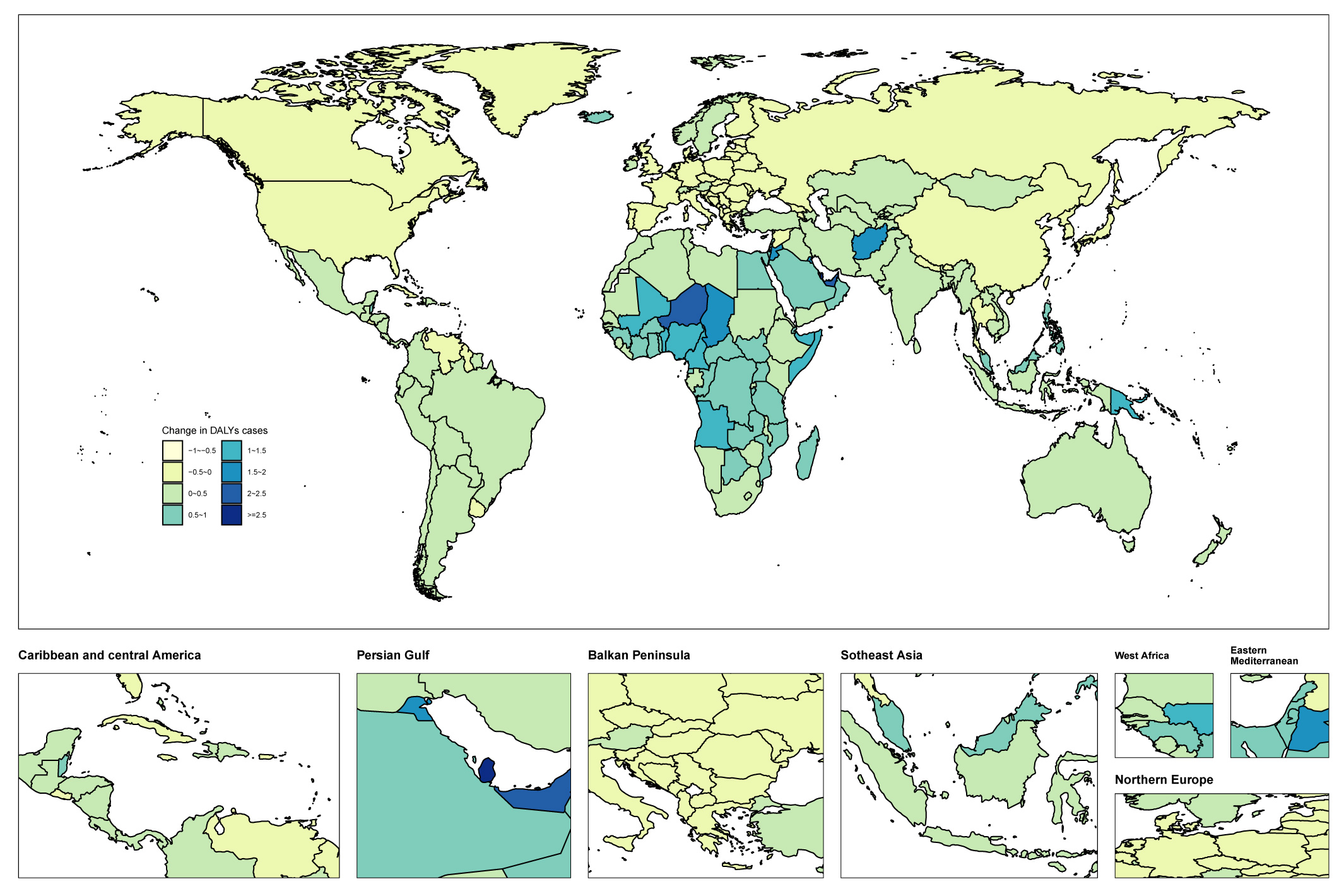


Supplementary Figure 5. Percentage change in DALYs cases across 204 countries in 1990 and 2021.


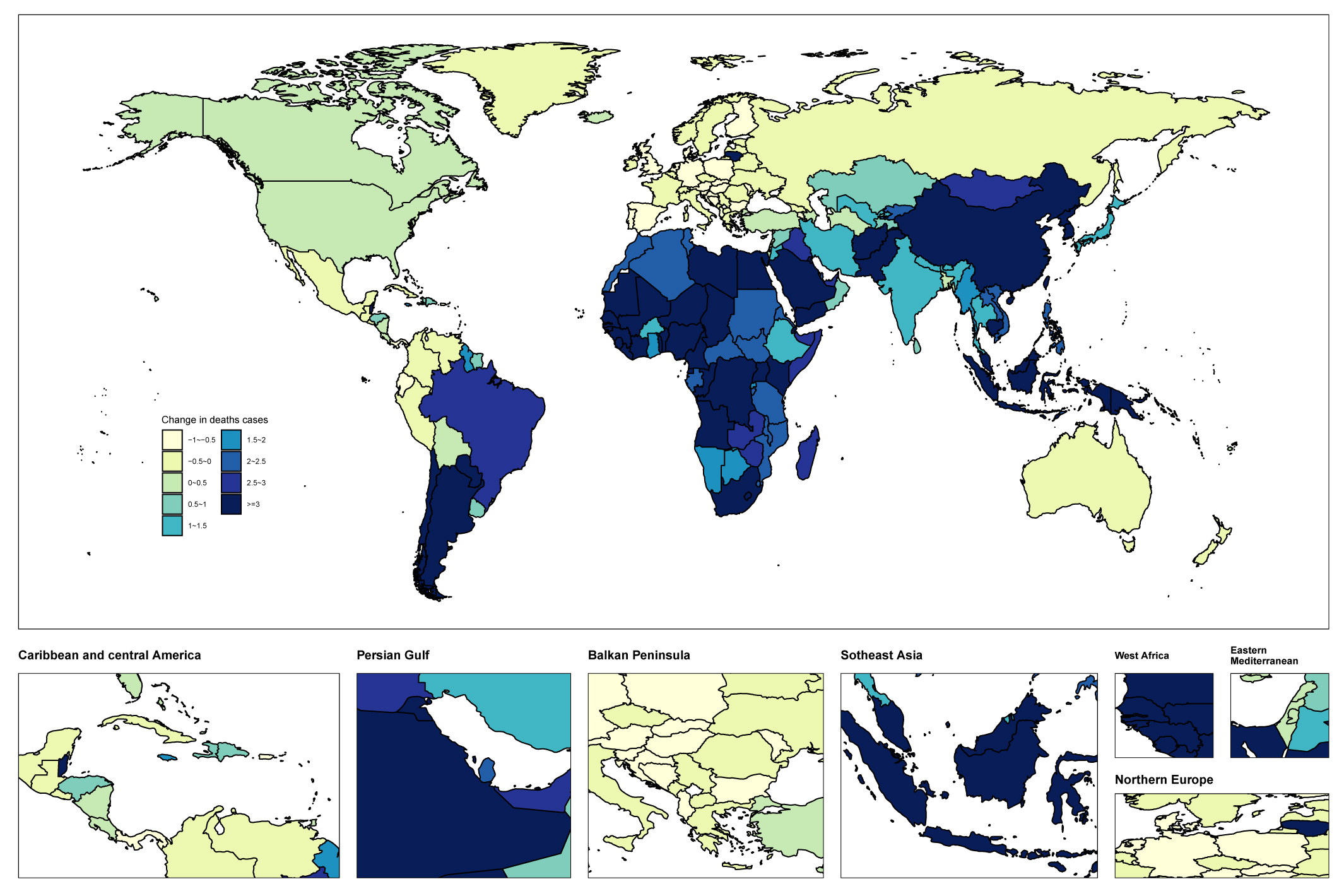


Supplementary Figure 6. Percentage change in death cases across 204 countries in 1990 and 2021.


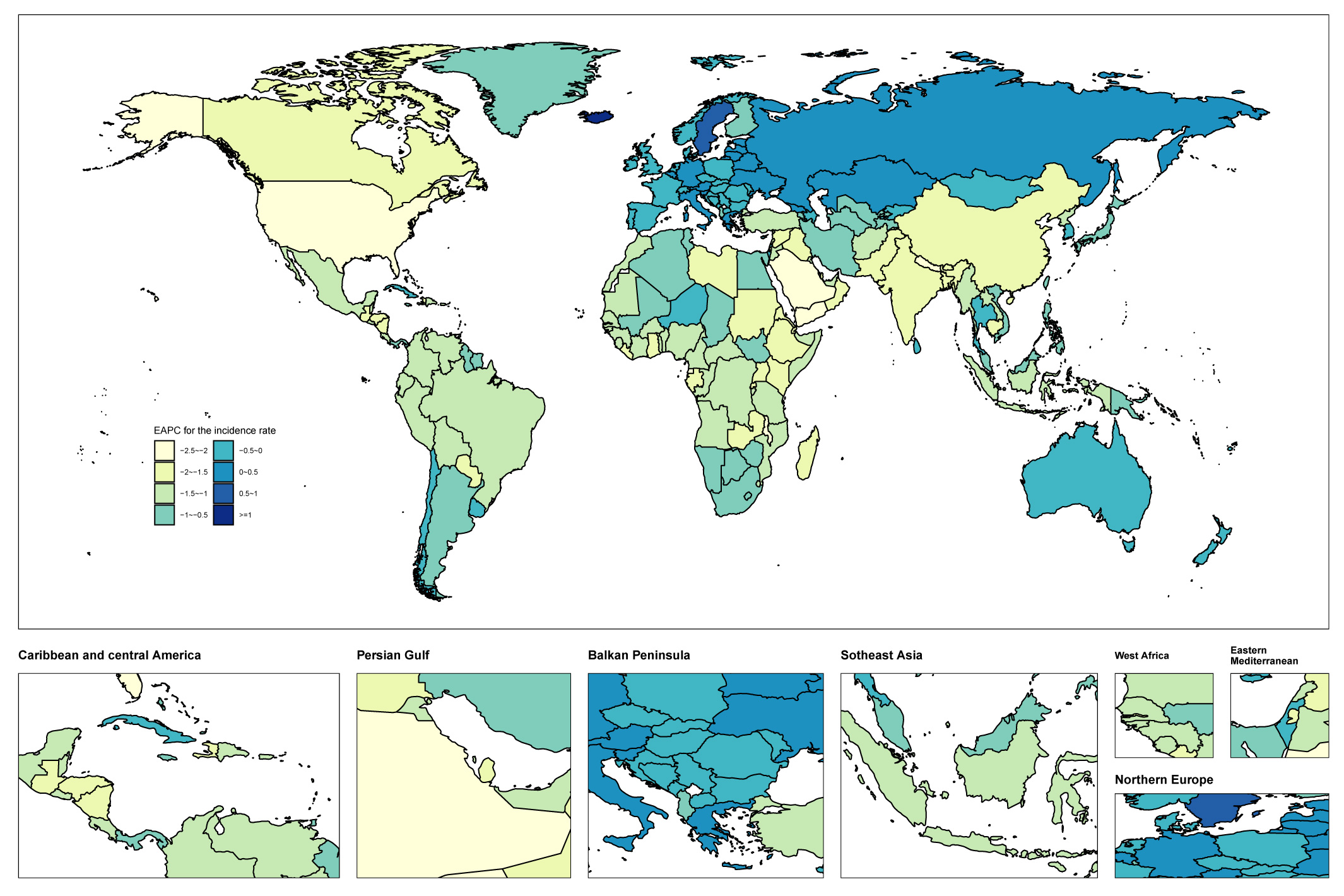


Supplementary Figure 7. EAPC in incidence rates across 204 countries from 1990 to 2021.


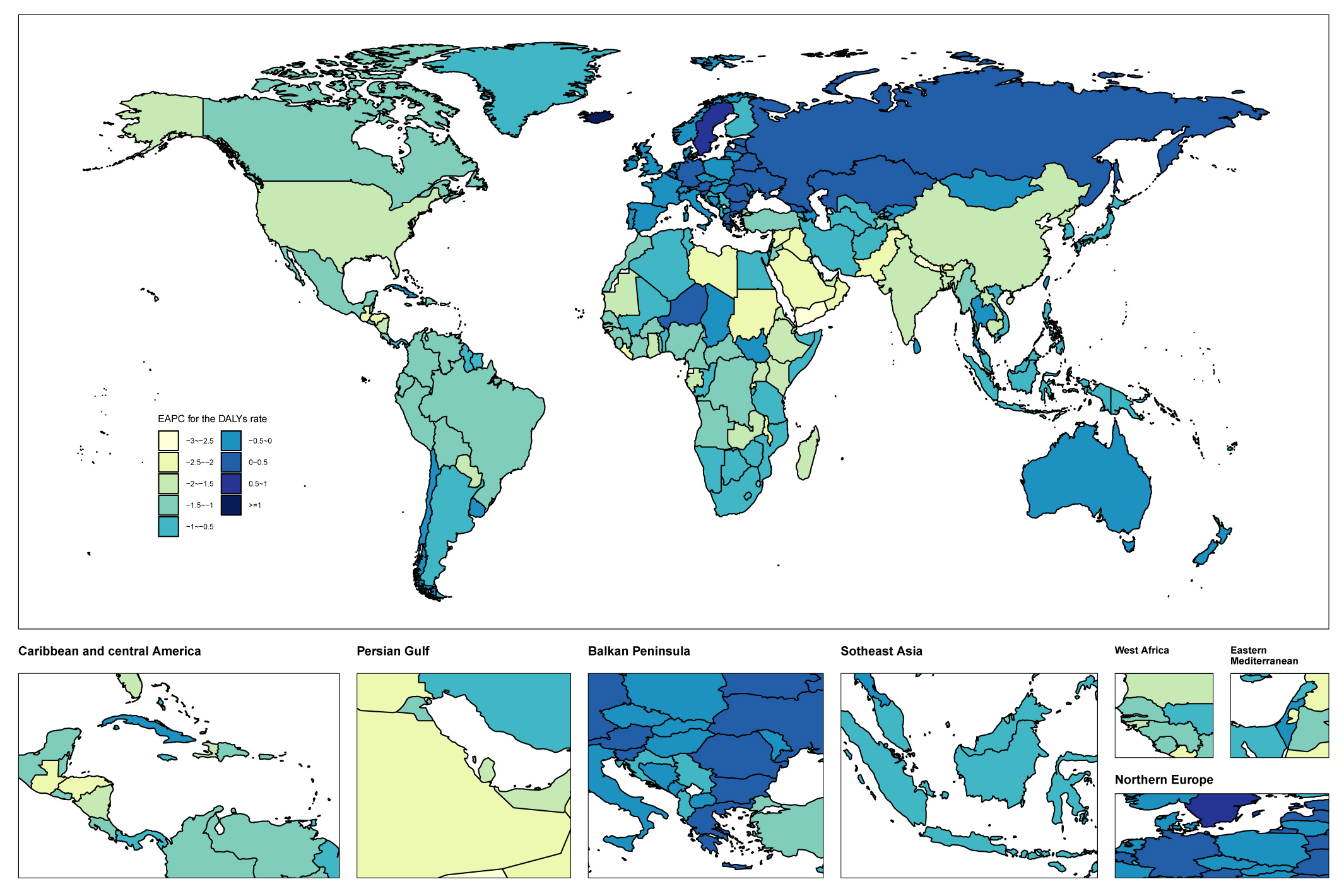
Supplementary Figure 8. EAPC in DALYs rates across 204 countries from 1990 to 2021.


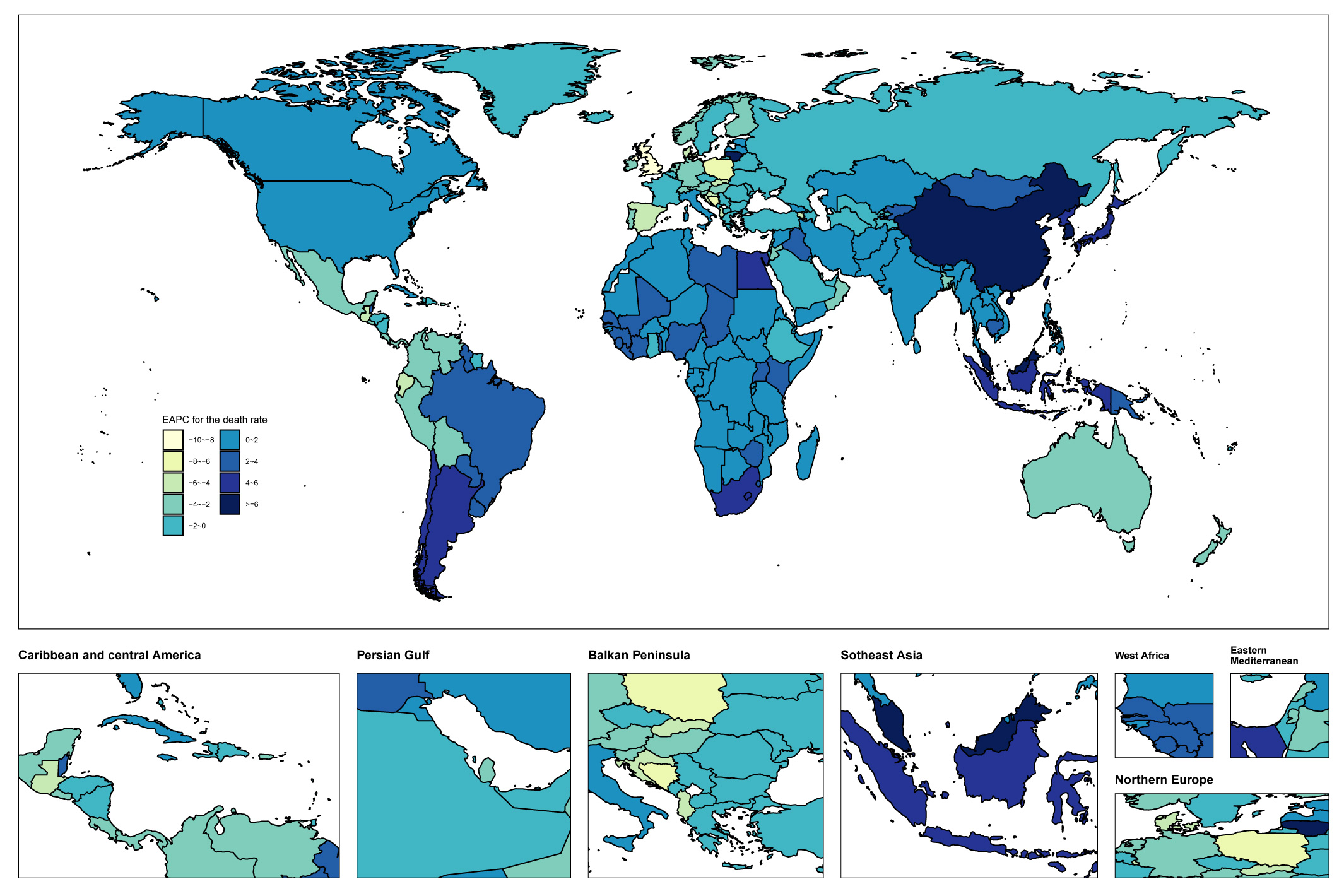


Supplementary Figure 9. EAPC in death rates across 204 countries from 1990 to 2021.


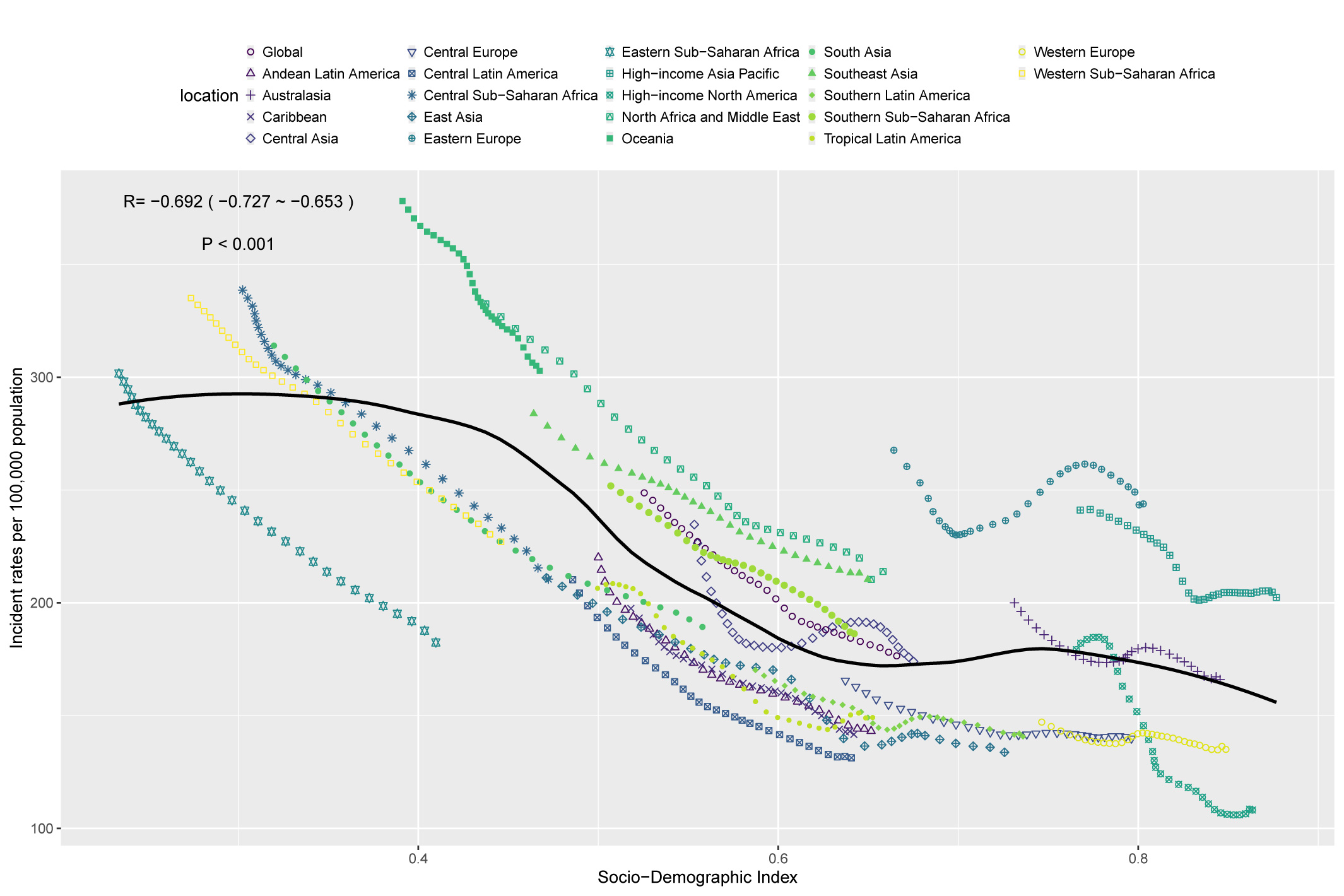


Supplementary Figure 10. The associations between the SDI and incidence rates per 100,000 population of endometriosis in WCBA across 21 GBD regions.


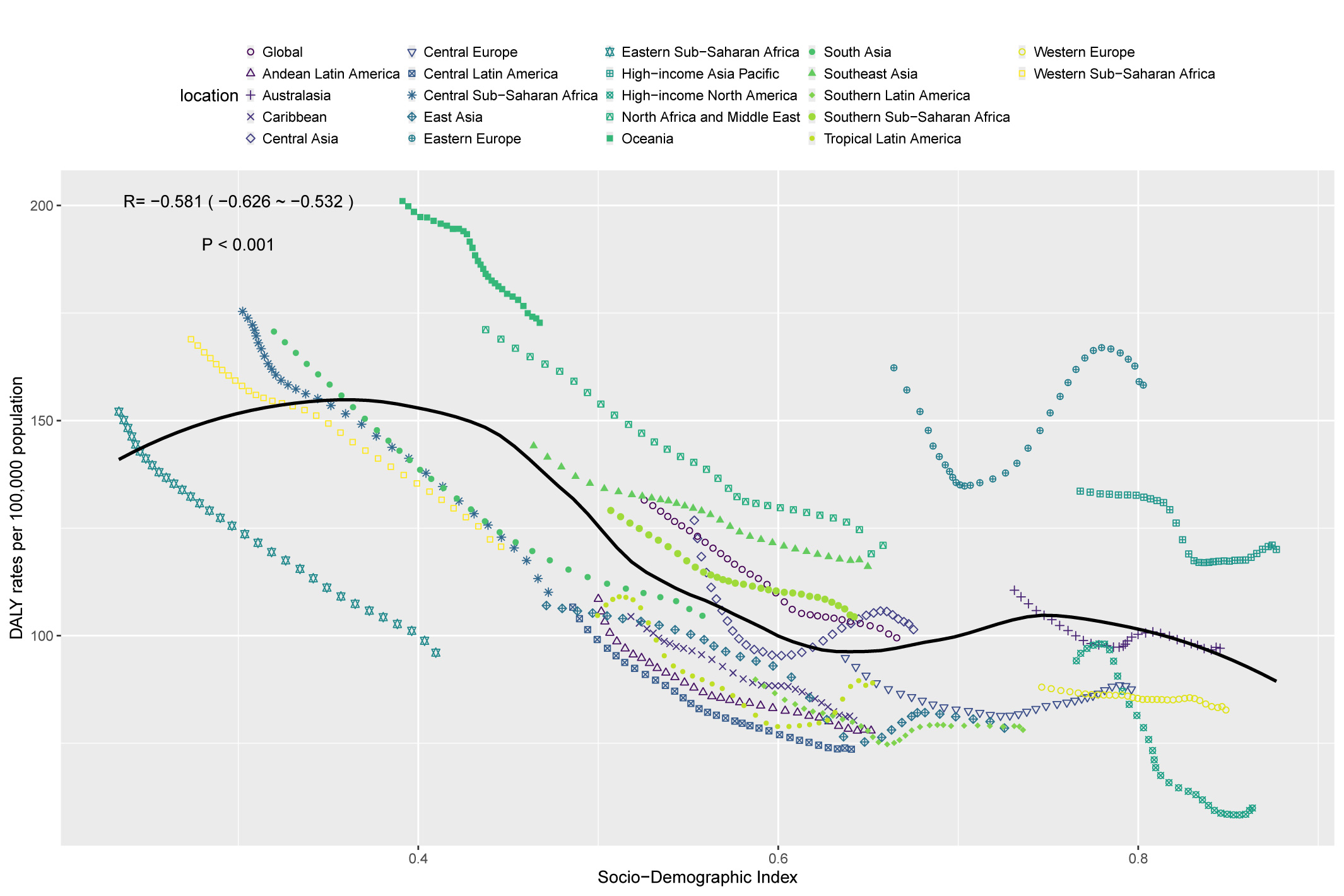
Supplementary Figure 11. The associations between the SDI and DALYs rates per 100,000 population of endometriosis in WCBA across 21 GBD regions.


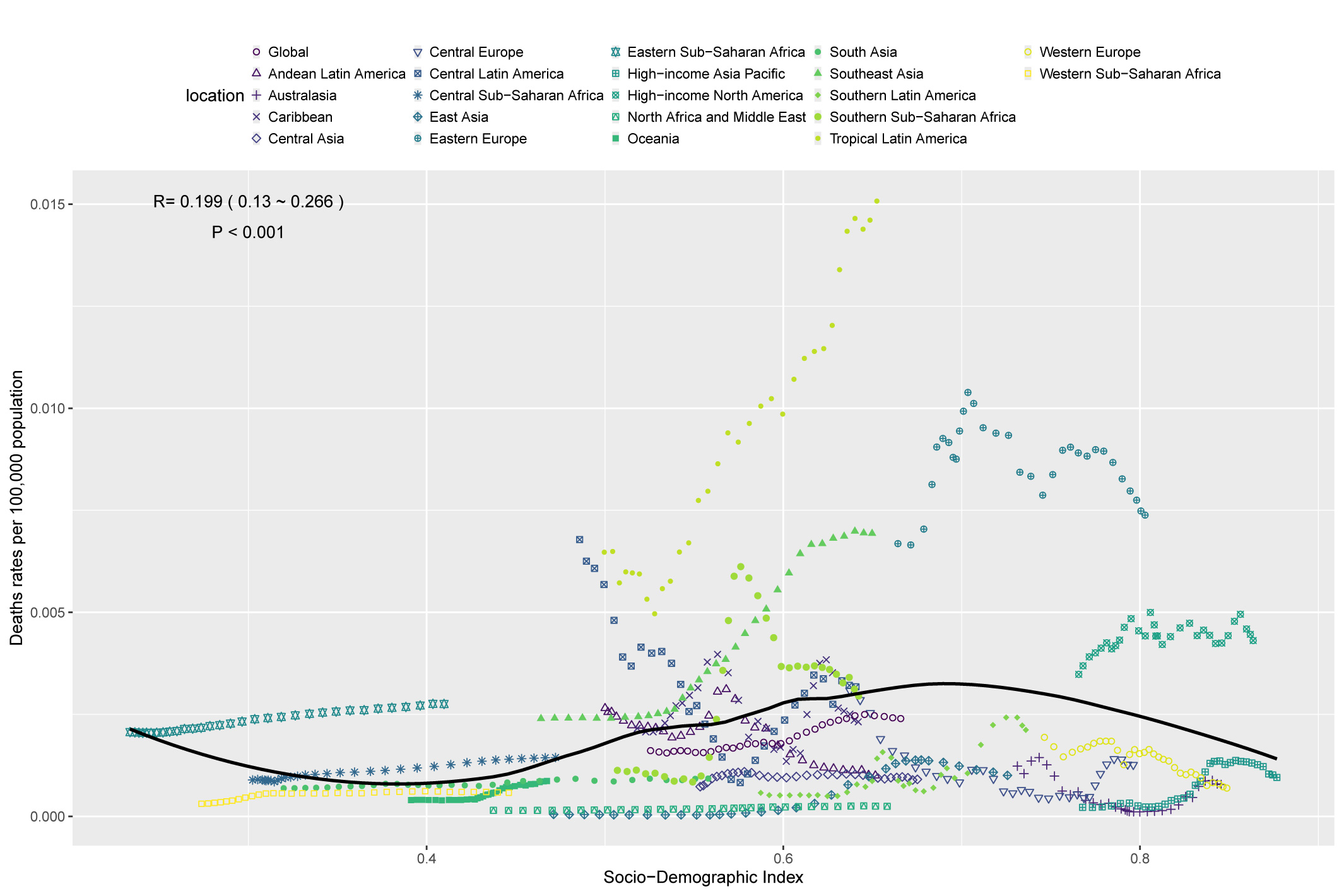


Supplementary Figure 12. The associations between the SDI and death rates per 100,000 population of endometriosis in WCBA across 21 GBD regions.
